# 5-Chloro-5′′-(4-chloro­benzyl­idene)-4′-(4-chloro­phen­yl)-1′′-ethyl-1′-methyl­dispiro­[indoline-3,2′-pyrrolidine-3′,3′′-piperidine]-2,4′′-dione

**DOI:** 10.1107/S1600536813033096

**Published:** 2013-12-14

**Authors:** I. S. Ahmed Farag, Adel S. Girgis, A. A. Ramadan, A. M. Moustafa, Edward R. T. Tiekink

**Affiliations:** a Solid State Department, Physics Division, National Research Centre, Dokki, Giza, Egypt; b Pesticide Chemistry Department, National Research Centre, Dokki, Giza 12622, Egypt; c Physics Department, Faculty of Science, Helwan University, Helwan, Cairo, Egypt; d Department of Chemistry, University of Malaya, 50603 Kuala Lumpur, Malaysia

## Abstract

Two spiro links are found in the title compound, C_31_H_28_Cl_3_N_3_O_2_, one connecting the piperidine and pyrrolidine rings, and the other connecting the pyrrolidine ring and indole residue. The piperidine ring adopts a half-chair conformation, in which the C atom connected to the spiro-C atom lies 0.741 (3) Å out of the plane of the remaining five atoms (r.m.s. deviation = 0.053 Å). The pyrrolidine ring has an envelope conformation with the flap atom being the methyl­ene C atom. Centrosymmetric eight-membered {⋯HNCO}_2_ amide dimers are the most significant feature of the crystal packing. These are connected into layers parallel to (-120) by C—H⋯O and π–π inter­actions between pyrrolidine-bound benzene rings [inter-centroid distance = 3.8348 (15) Å]. Slipped face-to-face inter­actions between the edges of pyrrolidine-bound benzene [shortest C⋯C separation = 3.484 (4) Å] connect the layers into a three-dimensional architecture.

## Related literature   

For the biological activity of related spiro­pyrrolidine analogues, see: Girgis *et al.* (2012 ▶); Kumar *et al.* (2008 ▶). For related structural studies, see: Farag *et al.* (2013 ▶). For the synthesis of the precursor mol­ecule, see Al-Omary *et al.* (2012 ▶).
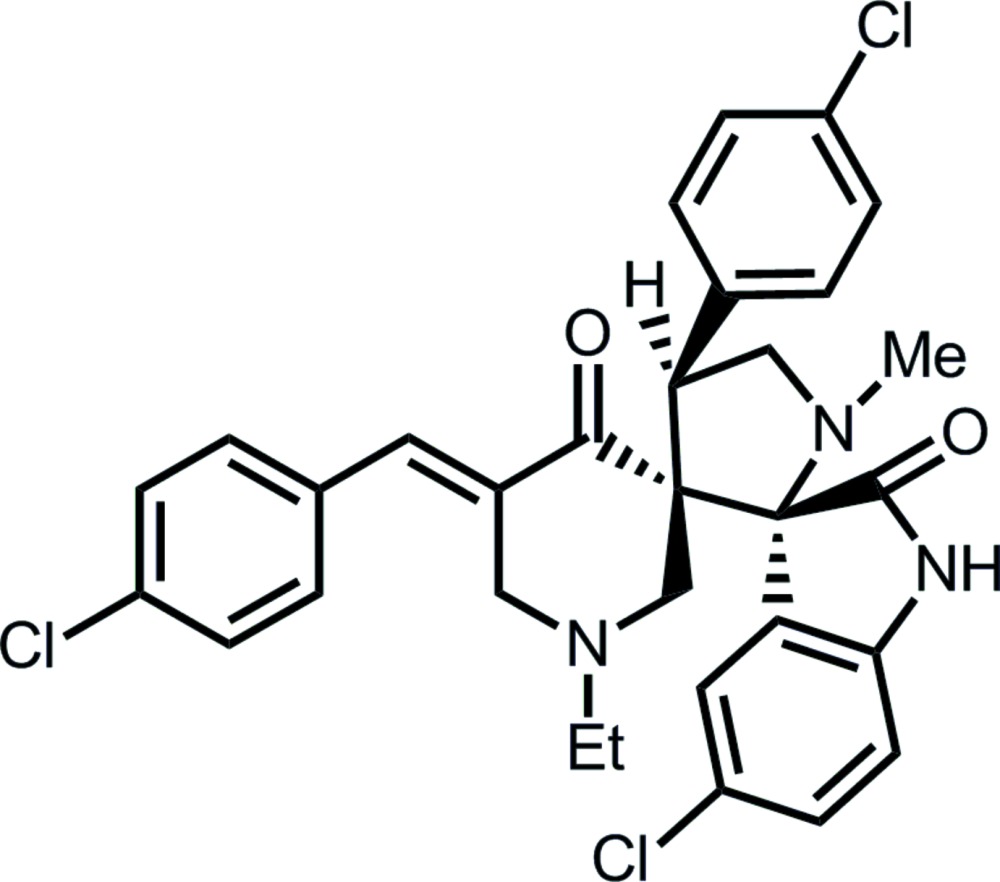


## Experimental   

### 

#### Crystal data  

C_31_H_28_Cl_3_N_3_O_2_
*M* *_r_* = 580.91
Triclinic, 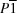

*a* = 11.1901 (2) Å
*b* = 11.6434 (3) Å
*c* = 12.4270 (3) Å
α = 99.477 (2)°
β = 90.235 (2)°
γ = 114.893 (1)°
*V* = 1443.77 (6) Å^3^
*Z* = 2
Mo *K*α radiation
μ = 0.35 mm^−1^
*T* = 293 K
1.02 × 0.53 × 0.37 mm

#### Data collection  

Nonius 590 KappaCCD diffractometer
Absorption correction: multi-scan (*SADABS*; Sheldrick, 1996 ▶) *T*_min_ = 0.880, *T*_max_ = 0.994
11344 measured reflections
6482 independent reflections
4260 reflections with *I* > 2σ(*I*)
*R*_int_ = 0.043

#### Refinement  

*R*[*F* ^2^ > 2σ(*F* ^2^)] = 0.057
*wR*(*F* ^2^) = 0.160
*S* = 1.00
6482 reflections
354 parameters
H-atom parameters constrained
Δρ_max_ = 0.72 e Å^−3^
Δρ_min_ = −0.73 e Å^−3^

### 

Data collection: *COLLECT* (Hooft, 1998 ▶); cell refinement: *DENZO* (Otwinowski & Minor, 1997 ▶) and *COLLECT*; data reduction: *DENZO* and *COLLECT*; program(s) used to solve structure: *SHELXS97* (Sheldrick, 2008 ▶); program(s) used to refine structure: *SHELXL97* (Sheldrick, 2008 ▶); molecular graphics: *ORTEP-3 for Windows* (Farrugia, 2012 ▶) and *DIAMOND* (Brandenburg, 2006 ▶); software used to prepare material for publication: *publCIF* (Westrip, 2010 ▶).

## Supplementary Material

Crystal structure: contains datablock(s) general, I. DOI: 10.1107/S1600536813033096/hb7170sup1.cif

Structure factors: contains datablock(s) I. DOI: 10.1107/S1600536813033096/hb7170Isup2.hkl

Additional supporting information:  crystallographic information; 3D view; checkCIF report

## Figures and Tables

**Table 1:** Hydrogen-bond geometry (Å, °)

*D*—H⋯*A*	*D*—H	H⋯*A*	*D*⋯*A*	*D*—H⋯*A*
N3—H3*n*⋯O2^i^	0.86	2.03	2.883 (3)	170
C31—H31⋯O1^ii^	0.93	2.47	3.160 (4)	131

Symmetry codes: (i) 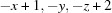; (ii) 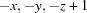.

**Fig. 1.**
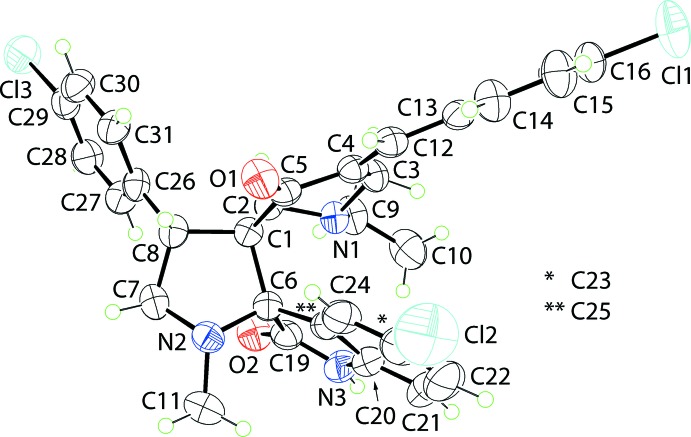
The molecular structure of (I) showing displacement ellipsoids at the 50% probability level.

**Fig. 2.**
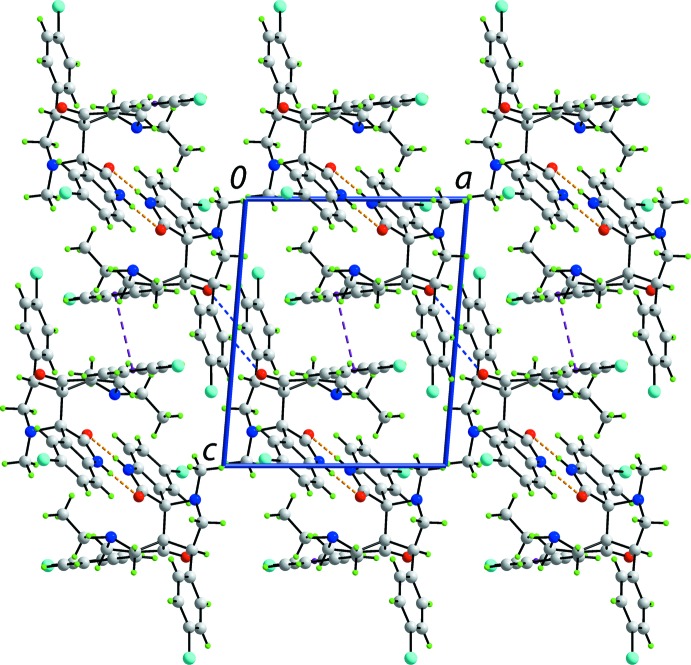
A view of the supramolecular layer in the ac plane in the crystal structure of (I). The N—H···O, C—H···O and π—π interactions are shown as orange, blue and purple dashed lines, respectively.

**Fig. 3.**
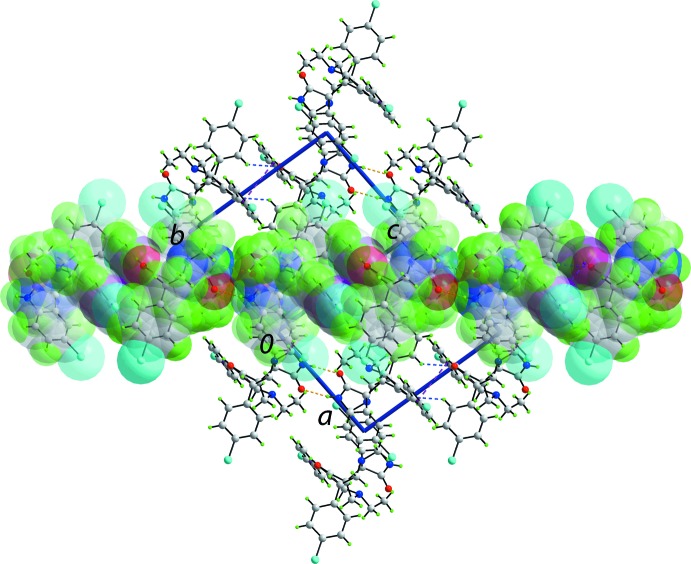
A view of the unit-cell contents in (I). One layer has been highlighted in space-filling mode. The N—H···O, C—H···O and π—π interactions are shown as orange, blue and purple dashed lines, respectively.

## supplementary crystallographic information

### 2.1. Synthesis and crystallization

A mixture of equimolar amounts of 3*E*,5*E*-1-ethyl-3,5-bis­({4-chloro­phenyl)­methyl­idene)-4-piperidone (5 mmol), prepared by a literature procedure (Al-Omary *et al.*, 2012), 5-chloro­isatin and sarcosine in absolute ethanol (25 ml) was boiled under reflux (TLC monitoring). The separated solid was collected and crystallized from *n*-butanol affording (I) as pale-yellow blocks. Reaction time 9 h. *M*.pt: 494–496 K. Yield 76%. Anal. Calcd. for C_31_H_28_Cl_3_N_3_O_2_ (580.95): C, 64.09; H, 4.86; N, 7.23. Found: C, 64.28; H, 5.02; N, 7.31. IR: ν_max_/cm^-1^: 3167 (N—H); 1689 (C═O); 1591, 1480 (C═C).

### 2.2. Refinement

The C-bound H atoms were geometrically placed (C—H = 0.93–0.98 Å) and refined as riding with *U_iso_*(H) = 1.2–1.5*U_eq_*(C). The N-bound H-atom was treated similarly with N—H = 0.86 Å, and with *U_iso_*(H) = 1.2*U_eq_*(N).

### 3. Results and discussion

Spiro­pyrrolidine derivatives are known to have biological activity and the basic sketal structure has been well established by X-ray crystallography (Kumar *et al.* 2008). In continuation of our biological and crystallographic studies of such derivatives (Girgis *et al.* 2012; Farag *et al.* 2013), the title compound, (I), was synthesised and characterised crystallographically.

Two spiro links exist in (I), Fig. 1, namely where the piperidine and pyrrolidine rings are connected at C1, and where the pyrrolidine ring and indole residue are connected at C6. The phenyl­methyl­idene and pyrrolidine-bound aryl residues are connected to the piperidine ring at positions C4 and C8, respectively. The conformation about the C4═C11 double bond is *E*. The *sp*^3^ character of the piperidine-N1 atom is confirmed by the sum of the angles about this atom, *i.e*. 335 °. The piperidine ring adopts a half-chair conformation where the C2 atom lies 0.741 (3) Å out of the plane of the remaining five atoms (r.m.s. deviation = 0.0527 Å). The C6 and C8 atoms occupy axial and equatorial positions with respect to the piperidine ring, the phenyl­methyl­idene residue occupies an equatorial position, and the N-bound methyl substituent is equatorial. The pyrrolidine ring has an envelope conformation with the flap atom being the C7 atom which lies 0.607 (4) Å out of the plane of the remaining four atoms (r.m.s. deviation = 0.0536 Å).

Centrosymmetric eight-membered {···HNCO}_2_ synthons are found in the crystal structure of (I), Table 1. The carbonyl-O1 atom also participates in other significant inter­molecular inter­actions, forming (pyrrolidine-bound benzene)C—H···O1 inter­actions with centrosymmetrically related dimers to form 14-membered {···HC_5_O}_2_ synthons leading to supra­molecular chains, Table 1. The chains are connected into a layer approximately parallel to (-1 2 0) by π—π, face-to-face, inter­actions [inter-centroid distance = 3.8348 (15) Å for symmetry operation: 1-*x*, 1-*y*, 1-*z*] between centrosymmetrically related methyl­idene-benzene rings (Fig. 2). The closest inter­actions between layers appear to be slipped face-to-face inter­actions between the edges (atoms C28 and C29) of pyrrolidine-bound benzene rings with the shortest separation being 3.484 (4) Å for C28···C28^i^ (symmetry operation *i*: -*x*, -1-*z*, 1-*z*); see Fig. 3.

### Crystal data

**Table:**

C_31_H_28_Cl_3_N_3_O_2_	*Z* = 2
*M**_r_* = 580.91	*F*(000) = 604
Triclinic, *P*1	*D*_x_ = 1.336 Mg m^−^^3^
Hall symbol: -P 1	Mo *K*α radiation, λ = 0.71073 Å
*a* = 11.1901 (2) Å	Cell parameters from 7513 reflections
*b* = 11.6434 (3) Å	θ = 2.9–27.9°
*c* = 12.4270 (3) Å	µ = 0.35 mm^−^^1^
α = 99.477 (2)°	*T* = 293 K
β = 90.235 (2)°	Block, pale-yellow
γ = 114.893 (1)°	1.02 × 0.53 × 0.37 mm
*V* = 1443.77 (6) Å^3^	

### Data collection

**Table:**

Nonius 590 KappaCCD diffractometer	6482 independent reflections
Radiation source: fine-focus sealed tube	4260 reflections with *I* > 2σ(*I*)
Graphite monochromator	*R*_int_ = 0.043
φ and ω scans	θ_max_ = 27.5°, θ_min_ = 3.3°
Absorption correction: multi-scan (*SADABS*; Sheldrick, 1996)	*h* = −12→14
*T*_min_ = 0.880, *T*_max_ = 0.994	*k* = −15→14
11344 measured reflections	*l* = −16→15

### Refinement

**Table:**

Refinement on *F*^2^	Primary atom site location: structure-invariant direct methods
Least-squares matrix: full	Secondary atom site location: difference Fourier map
*R*[*F*^2^ > 2σ(*F*^2^)] = 0.057	Hydrogen site location: inferred from neighbouring sites
*wR*(*F*^2^) = 0.160	H-atom parameters constrained
*S* = 1.00	*w* = 1/[σ^2^(*F*_o_^2^) + (0.0632*P*)^2^ + 0.8473*P*] where *P* = (*F*_o_^2^ + 2*F*_c_^2^)/3
6482 reflections	(Δ/σ)_max_ < 0.001
354 parameters	Δρ_max_ = 0.72 e Å^−^^3^
0 restraints	Δρ_min_ = −0.73 e Å^−^^3^

### Special details

**Table:**

Geometry. All s.u.'s (except the s.u. in the dihedral angle between two l.s. planes) are estimated using the full covariance matrix. The cell s.u.'s are taken into account individually in the estimation of s.u.'s in distances, angles and torsion angles; correlations between s.u.'s in cell parameters are only used when they are defined by crystal symmetry. An approximate (isotropic) treatment of cell s.u.'s is used for estimating s.u.'s involving l.s. planes.
Refinement. Refinement of *F*^2^ against ALL reflections. The weighted *R*-factor *wR* and goodness of fit *S* are based on *F*^2^, conventional *R*-factors *R* are based on *F*, with *F* set to zero for negative *F*^2^. The threshold expression of *F*^2^ > 2σ(*F*^2^) is used only for calculating *R*-factors(gt) *etc*. and is not relevant to the choice of reflections for refinement. *R*-factors based on *F*^2^ are statistically about twice as large as those based on *F*, and *R*- factors based on ALL data will be even larger.

### Fractional atomic coordinates and isotropic or equivalent isotropic displacement parameters (Å^2^)

**Table:**

	*x*	*y*	*z*	*U*_iso_*/*U*_eq_	
Cl1	0.75652 (9)	0.88544 (7)	0.62569 (9)	0.0843 (3)	
Cl2	0.18456 (16)	0.46678 (12)	0.97683 (11)	0.1288 (5)	
Cl3	0.08381 (9)	−0.48680 (8)	0.27827 (7)	0.0746 (3)	
O1	0.13605 (17)	0.13409 (17)	0.65765 (16)	0.0535 (5)	
O2	0.37169 (18)	−0.08975 (17)	0.88375 (15)	0.0527 (5)	
N1	0.48196 (18)	0.11736 (18)	0.73216 (16)	0.0388 (4)	
N2	0.11620 (19)	−0.05244 (19)	0.87264 (16)	0.0433 (5)	
N3	0.4280 (2)	0.1109 (2)	0.98503 (17)	0.0524 (5)	
H3n	0.4902	0.1139	1.0278	0.063*	
C1	0.2438 (2)	0.0150 (2)	0.72095 (18)	0.0352 (5)	
C2	0.3678 (2)	0.0046 (2)	0.67812 (19)	0.0388 (5)	
H2A	0.3713	−0.0728	0.6938	0.047*	
H2B	0.3663	0.0006	0.5995	0.047*	
C3	0.4971 (2)	0.2310 (2)	0.6878 (2)	0.0427 (5)	
H3A	0.5315	0.2262	0.6165	0.051*	
H3B	0.5614	0.3072	0.7354	0.051*	
C4	0.3699 (2)	0.2447 (2)	0.67642 (18)	0.0381 (5)	
C5	0.2403 (2)	0.1327 (2)	0.68197 (18)	0.0375 (5)	
C6	0.2491 (2)	0.0382 (2)	0.85091 (18)	0.0370 (5)	
C7	0.0761 (3)	−0.1661 (2)	0.7874 (2)	0.0485 (6)	
H7A	−0.0180	−0.2201	0.7849	0.058*	
H7B	0.1242	−0.2164	0.7981	0.058*	
C8	0.1119 (2)	−0.1086 (2)	0.68402 (19)	0.0407 (5)	
H8	0.0449	−0.0790	0.6682	0.049*	
C9	0.6045 (3)	0.0993 (3)	0.7295 (3)	0.0542 (7)	
H9A	0.6395	0.1114	0.6589	0.065*	
H9B	0.5841	0.0116	0.7373	0.065*	
C10	0.7081 (3)	0.1904 (3)	0.8178 (3)	0.0718 (9)	
H10A	0.7305	0.2774	0.8095	0.108*	
H10B	0.7854	0.1743	0.8125	0.108*	
H10C	0.6747	0.1778	0.8880	0.108*	
C11	0.1021 (3)	−0.0799 (3)	0.9836 (2)	0.0609 (7)	
H11A	0.0106	−0.1300	0.9925	0.091*	
H11B	0.1349	−0.0004	1.0355	0.091*	
H11C	0.1517	−0.1274	0.9959	0.091*	
C12	0.3628 (3)	0.3537 (2)	0.66025 (19)	0.0424 (5)	
H12	0.2769	0.3464	0.6538	0.051*	
C13	0.4638 (2)	0.4803 (2)	0.65098 (18)	0.0405 (5)	
C14	0.4248 (3)	0.5800 (3)	0.6556 (2)	0.0534 (7)	
H14	0.3366	0.5627	0.6642	0.064*	
C15	0.5127 (3)	0.7033 (3)	0.6478 (3)	0.0611 (8)	
H15	0.4839	0.7679	0.6510	0.073*	
C16	0.6433 (3)	0.7303 (2)	0.6353 (2)	0.0537 (7)	
C17	0.6857 (3)	0.6346 (3)	0.6289 (2)	0.0538 (7)	
H17	0.7740	0.6528	0.6198	0.065*	
C18	0.5963 (3)	0.5111 (2)	0.6361 (2)	0.0497 (6)	
H18	0.6255	0.4466	0.6309	0.060*	
C19	0.3588 (2)	0.0111 (2)	0.9046 (2)	0.0441 (6)	
C20	0.3866 (3)	0.2094 (3)	0.9908 (2)	0.0519 (6)	
C21	0.4321 (4)	0.3248 (3)	1.0627 (3)	0.0784 (10)	
H21	0.5032	0.3489	1.1138	0.094*	
C22	0.3694 (5)	0.4044 (3)	1.0570 (3)	0.0883 (12)	
H22	0.3992	0.4836	1.1040	0.106*	
C23	0.2635 (4)	0.3662 (3)	0.9821 (3)	0.0741 (10)	
C24	0.2167 (3)	0.2502 (3)	0.9093 (2)	0.0527 (7)	
H24	0.1443	0.2256	0.8594	0.063*	
C25	0.2810 (2)	0.1722 (2)	0.91300 (19)	0.0427 (6)	
C26	0.1113 (2)	−0.2007 (2)	0.5831 (2)	0.0407 (5)	
C27	0.1627 (3)	−0.2913 (2)	0.5846 (2)	0.0485 (6)	
H27	0.2025	−0.2933	0.6496	0.058*	
C28	0.1555 (3)	−0.3778 (2)	0.4911 (2)	0.0518 (6)	
H28	0.1906	−0.4371	0.4932	0.062*	
C29	0.0960 (3)	−0.3750 (2)	0.3951 (2)	0.0501 (6)	
C30	0.0446 (3)	−0.2871 (3)	0.3903 (2)	0.0530 (6)	
H30	0.0049	−0.2858	0.3249	0.064*	
C31	0.0529 (3)	−0.2008 (2)	0.4842 (2)	0.0479 (6)	
H31	0.0184	−0.1412	0.4810	0.057*	

### Atomic displacement parameters (Å^2^)

**Table:**

	*U*^11^	*U*^22^	*U*^33^	*U*^12^	*U*^13^	*U*^23^
Cl1	0.0726 (5)	0.0396 (4)	0.1372 (8)	0.0211 (4)	0.0221 (5)	0.0141 (4)
Cl2	0.1991 (14)	0.0931 (8)	0.1389 (10)	0.1089 (9)	0.0425 (9)	0.0100 (7)
Cl3	0.0954 (6)	0.0525 (4)	0.0640 (5)	0.0258 (4)	0.0110 (4)	−0.0047 (3)
O1	0.0433 (10)	0.0552 (11)	0.0732 (12)	0.0273 (9)	−0.0027 (9)	0.0251 (9)
O2	0.0540 (11)	0.0530 (11)	0.0630 (11)	0.0307 (9)	−0.0014 (9)	0.0207 (9)
N1	0.0349 (10)	0.0363 (10)	0.0505 (11)	0.0202 (9)	0.0005 (8)	0.0086 (9)
N2	0.0421 (11)	0.0459 (12)	0.0451 (11)	0.0204 (10)	0.0071 (9)	0.0121 (9)
N3	0.0534 (13)	0.0570 (14)	0.0457 (12)	0.0222 (11)	−0.0104 (10)	0.0108 (11)
C1	0.0361 (12)	0.0351 (11)	0.0389 (12)	0.0194 (10)	0.0001 (9)	0.0066 (9)
C2	0.0422 (13)	0.0370 (12)	0.0413 (13)	0.0218 (11)	0.0008 (10)	0.0048 (10)
C3	0.0439 (13)	0.0359 (12)	0.0521 (14)	0.0196 (11)	0.0036 (11)	0.0113 (11)
C4	0.0454 (13)	0.0388 (12)	0.0351 (12)	0.0223 (11)	0.0018 (10)	0.0087 (10)
C5	0.0426 (13)	0.0408 (12)	0.0340 (11)	0.0224 (11)	−0.0001 (9)	0.0074 (10)
C6	0.0375 (12)	0.0370 (12)	0.0397 (12)	0.0182 (10)	0.0004 (9)	0.0091 (10)
C7	0.0411 (14)	0.0407 (13)	0.0587 (16)	0.0120 (11)	0.0032 (11)	0.0114 (12)
C8	0.0367 (12)	0.0389 (12)	0.0485 (14)	0.0185 (10)	−0.0033 (10)	0.0064 (10)
C9	0.0404 (14)	0.0482 (15)	0.0819 (19)	0.0249 (12)	0.0036 (13)	0.0162 (14)
C10	0.0414 (16)	0.083 (2)	0.094 (2)	0.0232 (16)	0.0001 (15)	0.0325 (19)
C11	0.0635 (18)	0.0696 (19)	0.0572 (17)	0.0301 (16)	0.0193 (14)	0.0268 (15)
C12	0.0490 (14)	0.0449 (13)	0.0405 (13)	0.0261 (12)	0.0026 (10)	0.0102 (11)
C13	0.0508 (14)	0.0392 (12)	0.0368 (12)	0.0242 (11)	−0.0013 (10)	0.0072 (10)
C14	0.0502 (15)	0.0458 (15)	0.0723 (18)	0.0274 (13)	0.0013 (13)	0.0133 (13)
C15	0.0601 (18)	0.0401 (15)	0.089 (2)	0.0278 (14)	0.0019 (15)	0.0099 (14)
C16	0.0580 (17)	0.0357 (13)	0.0656 (17)	0.0194 (12)	0.0047 (13)	0.0063 (12)
C17	0.0545 (16)	0.0475 (15)	0.0666 (17)	0.0278 (13)	0.0118 (13)	0.0129 (13)
C18	0.0603 (16)	0.0436 (14)	0.0569 (16)	0.0315 (13)	0.0118 (13)	0.0141 (12)
C19	0.0434 (14)	0.0485 (14)	0.0446 (14)	0.0207 (12)	0.0012 (11)	0.0164 (12)
C20	0.0623 (17)	0.0486 (15)	0.0371 (13)	0.0167 (13)	0.0002 (12)	0.0064 (11)
C21	0.105 (3)	0.062 (2)	0.0463 (17)	0.020 (2)	−0.0093 (17)	−0.0034 (15)
C22	0.141 (4)	0.054 (2)	0.057 (2)	0.036 (2)	0.013 (2)	−0.0079 (16)
C23	0.118 (3)	0.0525 (18)	0.063 (2)	0.047 (2)	0.028 (2)	0.0078 (15)
C24	0.0703 (18)	0.0499 (15)	0.0479 (15)	0.0344 (14)	0.0157 (13)	0.0105 (12)
C25	0.0527 (15)	0.0417 (13)	0.0361 (12)	0.0216 (12)	0.0068 (10)	0.0086 (10)
C26	0.0357 (12)	0.0341 (12)	0.0507 (14)	0.0142 (10)	−0.0038 (10)	0.0058 (10)
C27	0.0516 (15)	0.0440 (14)	0.0542 (15)	0.0248 (12)	−0.0038 (12)	0.0089 (12)
C28	0.0580 (16)	0.0399 (14)	0.0613 (17)	0.0250 (13)	0.0053 (13)	0.0077 (12)
C29	0.0508 (15)	0.0379 (13)	0.0527 (15)	0.0122 (12)	0.0064 (12)	0.0033 (11)
C30	0.0546 (16)	0.0544 (16)	0.0486 (15)	0.0230 (13)	−0.0057 (12)	0.0068 (12)
C31	0.0478 (14)	0.0469 (14)	0.0524 (15)	0.0241 (12)	−0.0056 (11)	0.0079 (12)

### Geometric parameters (Å, º)

**Table:**

Cl1—C16	1.740 (3)	C10—H10B	0.9600
Cl2—C23	1.747 (3)	C10—H10C	0.9600
Cl3—C29	1.745 (3)	C11—H11A	0.9600
O1—C5	1.211 (3)	C11—H11B	0.9600
O2—C19	1.229 (3)	C11—H11C	0.9600
N1—C2	1.448 (3)	C12—C13	1.456 (3)
N1—C3	1.461 (3)	C12—H12	0.9300
N1—C9	1.472 (3)	C13—C18	1.392 (4)
N2—C7	1.453 (3)	C13—C14	1.395 (3)
N2—C11	1.461 (3)	C14—C15	1.377 (4)
N2—C6	1.475 (3)	C14—H14	0.9300
N3—C19	1.343 (3)	C15—C16	1.375 (4)
N3—C20	1.398 (3)	C15—H15	0.9300
N3—H3n	0.8600	C16—C17	1.375 (4)
C1—C2	1.532 (3)	C17—C18	1.382 (4)
C1—C5	1.543 (3)	C17—H17	0.9300
C1—C8	1.565 (3)	C18—H18	0.9300
C1—C6	1.589 (3)	C20—C21	1.373 (4)
C2—H2A	0.9700	C20—C25	1.393 (4)
C2—H2B	0.9700	C21—C22	1.387 (5)
C3—C4	1.506 (3)	C21—H21	0.9300
C3—H3A	0.9700	C22—C23	1.371 (5)
C3—H3B	0.9700	C22—H22	0.9300
C4—C12	1.351 (3)	C23—C24	1.384 (4)
C4—C5	1.499 (3)	C24—C25	1.381 (3)
C6—C25	1.514 (3)	C24—H24	0.9300
C6—C19	1.561 (3)	C26—C31	1.389 (3)
C7—C8	1.524 (3)	C26—C27	1.401 (3)
C7—H7A	0.9700	C27—C28	1.384 (4)
C7—H7B	0.9700	C27—H27	0.9300
C8—C26	1.508 (3)	C28—C29	1.376 (4)
C8—H8	0.9800	C28—H28	0.9300
C9—C10	1.498 (4)	C29—C30	1.378 (4)
C9—H9A	0.9700	C30—C31	1.384 (4)
C9—H9B	0.9700	C30—H30	0.9300
C10—H10A	0.9600	C31—H31	0.9300
			
C2—N1—C3	110.85 (18)	H11A—C11—H11B	109.5
C2—N1—C9	113.09 (19)	N2—C11—H11C	109.5
C3—N1—C9	111.41 (19)	H11A—C11—H11C	109.5
C7—N2—C11	114.2 (2)	H11B—C11—H11C	109.5
C7—N2—C6	106.79 (18)	C4—C12—C13	132.3 (2)
C11—N2—C6	115.8 (2)	C4—C12—H12	113.9
C19—N3—C20	111.8 (2)	C13—C12—H12	113.9
C19—N3—H3n	124.1	C18—C13—C14	116.6 (2)
C20—N3—H3n	124.1	C18—C13—C12	125.9 (2)
C2—C1—C5	106.00 (18)	C14—C13—C12	117.5 (2)
C2—C1—C8	114.65 (18)	C15—C14—C13	122.0 (3)
C5—C1—C8	111.33 (17)	C15—C14—H14	119.0
C2—C1—C6	112.13 (17)	C13—C14—H14	119.0
C5—C1—C6	108.72 (17)	C16—C15—C14	119.6 (2)
C8—C1—C6	103.98 (17)	C16—C15—H15	120.2
N1—C2—C1	107.91 (18)	C14—C15—H15	120.2
N1—C2—H2A	110.1	C15—C16—C17	120.3 (3)
C1—C2—H2A	110.1	C15—C16—Cl1	120.5 (2)
N1—C2—H2B	110.1	C17—C16—Cl1	119.2 (2)
C1—C2—H2B	110.1	C16—C17—C18	119.5 (3)
H2A—C2—H2B	108.4	C16—C17—H17	120.3
N1—C3—C4	113.32 (19)	C18—C17—H17	120.3
N1—C3—H3A	108.9	C17—C18—C13	122.0 (2)
C4—C3—H3A	108.9	C17—C18—H18	119.0
N1—C3—H3B	108.9	C13—C18—H18	119.0
C4—C3—H3B	108.9	O2—C19—N3	125.4 (2)
H3A—C3—H3B	107.7	O2—C19—C6	125.6 (2)
C12—C4—C5	115.8 (2)	N3—C19—C6	108.6 (2)
C12—C4—C3	124.2 (2)	C21—C20—C25	121.6 (3)
C5—C4—C3	120.06 (19)	C21—C20—N3	128.8 (3)
O1—C5—C4	121.7 (2)	C25—C20—N3	109.6 (2)
O1—C5—C1	120.7 (2)	C20—C21—C22	118.4 (3)
C4—C5—C1	117.58 (18)	C20—C21—H21	120.8
N2—C6—C25	110.00 (19)	C22—C21—H21	120.8
N2—C6—C19	111.18 (18)	C23—C22—C21	119.9 (3)
C25—C6—C19	100.62 (19)	C23—C22—H22	120.0
N2—C6—C1	103.23 (17)	C21—C22—H22	120.0
C25—C6—C1	118.74 (17)	C22—C23—C24	122.3 (3)
C19—C6—C1	113.26 (18)	C22—C23—Cl2	119.4 (3)
N2—C7—C8	102.53 (19)	C24—C23—Cl2	118.3 (3)
N2—C7—H7A	111.3	C25—C24—C23	117.9 (3)
C8—C7—H7A	111.3	C25—C24—H24	121.1
N2—C7—H7B	111.3	C23—C24—H24	121.1
C8—C7—H7B	111.3	C24—C25—C20	119.9 (2)
H7A—C7—H7B	109.2	C24—C25—C6	130.6 (2)
C26—C8—C7	115.4 (2)	C20—C25—C6	109.2 (2)
C26—C8—C1	117.06 (19)	C31—C26—C27	117.5 (2)
C7—C8—C1	103.79 (18)	C31—C26—C8	119.5 (2)
C26—C8—H8	106.6	C27—C26—C8	122.9 (2)
C7—C8—H8	106.6	C28—C27—C26	121.3 (2)
C1—C8—H8	106.6	C28—C27—H27	119.4
N1—C9—C10	112.9 (2)	C26—C27—H27	119.4
N1—C9—H9A	109.0	C29—C28—C27	119.2 (2)
C10—C9—H9A	109.0	C29—C28—H28	120.4
N1—C9—H9B	109.0	C27—C28—H28	120.4
C10—C9—H9B	109.0	C28—C29—C30	121.1 (2)
H9A—C9—H9B	107.8	C28—C29—Cl3	119.0 (2)
C9—C10—H10A	109.5	C30—C29—Cl3	119.8 (2)
C9—C10—H10B	109.5	C29—C30—C31	119.1 (2)
H10A—C10—H10B	109.5	C29—C30—H30	120.5
C9—C10—H10C	109.5	C31—C30—H30	120.5
H10A—C10—H10C	109.5	C30—C31—C26	121.8 (2)
H10B—C10—H10C	109.5	C30—C31—H31	119.1
N2—C11—H11A	109.5	C26—C31—H31	119.1
N2—C11—H11B	109.5		
			
C3—N1—C2—C1	−74.5 (2)	C13—C14—C15—C16	−0.1 (5)
C9—N1—C2—C1	159.54 (19)	C14—C15—C16—C17	1.0 (5)
C5—C1—C2—N1	65.3 (2)	C14—C15—C16—Cl1	−179.8 (2)
C8—C1—C2—N1	−171.45 (17)	C15—C16—C17—C18	−0.6 (4)
C6—C1—C2—N1	−53.2 (2)	Cl1—C16—C17—C18	−179.8 (2)
C2—N1—C3—C4	46.3 (3)	C16—C17—C18—C13	−0.7 (4)
C9—N1—C3—C4	173.2 (2)	C14—C13—C18—C17	1.5 (4)
N1—C3—C4—C12	165.1 (2)	C12—C13—C18—C17	−179.6 (2)
N1—C3—C4—C5	−15.4 (3)	C20—N3—C19—O2	−177.5 (2)
C12—C4—C5—O1	10.4 (3)	C20—N3—C19—C6	−3.7 (3)
C3—C4—C5—O1	−169.2 (2)	N2—C6—C19—O2	60.9 (3)
C12—C4—C5—C1	−169.1 (2)	C25—C6—C19—O2	177.3 (2)
C3—C4—C5—C1	11.3 (3)	C1—C6—C19—O2	−54.8 (3)
C2—C1—C5—O1	146.0 (2)	N2—C6—C19—N3	−112.9 (2)
C8—C1—C5—O1	20.7 (3)	C25—C6—C19—N3	3.6 (2)
C6—C1—C5—O1	−93.2 (3)	C1—C6—C19—N3	131.4 (2)
C2—C1—C5—C4	−34.5 (3)	C19—N3—C20—C21	179.0 (3)
C8—C1—C5—C4	−159.77 (19)	C19—N3—C20—C25	2.2 (3)
C6—C1—C5—C4	86.3 (2)	C25—C20—C21—C22	0.6 (5)
C7—N2—C6—C25	162.75 (18)	N3—C20—C21—C22	−175.8 (3)
C11—N2—C6—C25	−68.9 (3)	C20—C21—C22—C23	1.0 (5)
C7—N2—C6—C19	−86.7 (2)	C21—C22—C23—C24	−1.0 (5)
C11—N2—C6—C19	41.7 (3)	C21—C22—C23—Cl2	179.4 (3)
C7—N2—C6—C1	35.1 (2)	C22—C23—C24—C25	−0.5 (5)
C11—N2—C6—C1	163.4 (2)	Cl2—C23—C24—C25	179.1 (2)
C2—C1—C6—N2	−134.84 (18)	C23—C24—C25—C20	2.1 (4)
C5—C1—C6—N2	108.29 (19)	C23—C24—C25—C6	175.3 (3)
C8—C1—C6—N2	−10.4 (2)	C21—C20—C25—C24	−2.2 (4)
C2—C1—C6—C25	103.2 (2)	N3—C20—C25—C24	174.9 (2)
C5—C1—C6—C25	−13.7 (3)	C21—C20—C25—C6	−176.8 (3)
C8—C1—C6—C25	−132.4 (2)	N3—C20—C25—C6	0.3 (3)
C2—C1—C6—C19	−14.5 (3)	N2—C6—C25—C24	−58.7 (3)
C5—C1—C6—C19	−131.4 (2)	C19—C6—C25—C24	−176.1 (2)
C8—C1—C6—C19	109.9 (2)	C1—C6—C25—C24	59.8 (3)
C11—N2—C7—C8	−175.4 (2)	N2—C6—C25—C20	115.1 (2)
C6—N2—C7—C8	−46.1 (2)	C19—C6—C25—C20	−2.3 (2)
N2—C7—C8—C26	166.56 (19)	C1—C6—C25—C20	−126.4 (2)
N2—C7—C8—C1	37.1 (2)	C7—C8—C26—C31	135.5 (2)
C2—C1—C8—C26	−21.6 (3)	C1—C8—C26—C31	−101.9 (3)
C5—C1—C8—C26	98.7 (2)	C7—C8—C26—C27	−41.9 (3)
C6—C1—C8—C26	−144.36 (19)	C1—C8—C26—C27	80.7 (3)
C2—C1—C8—C7	106.9 (2)	C31—C26—C27—C28	−0.1 (4)
C5—C1—C8—C7	−132.80 (19)	C8—C26—C27—C28	177.3 (2)
C6—C1—C8—C7	−15.9 (2)	C26—C27—C28—C29	−0.4 (4)
C2—N1—C9—C10	−158.0 (2)	C27—C28—C29—C30	0.6 (4)
C3—N1—C9—C10	76.3 (3)	C27—C28—C29—Cl3	−178.5 (2)
C5—C4—C12—C13	179.6 (2)	C28—C29—C30—C31	−0.3 (4)
C3—C4—C12—C13	−0.9 (4)	Cl3—C29—C30—C31	178.8 (2)
C4—C12—C13—C18	13.4 (4)	C29—C30—C31—C26	−0.2 (4)
C4—C12—C13—C14	−167.7 (3)	C27—C26—C31—C30	0.4 (4)
C18—C13—C14—C15	−1.1 (4)	C8—C26—C31—C30	−177.2 (2)
C12—C13—C14—C15	179.9 (3)		

### Hydrogen-bond geometry (Å, º)

**Table:**

*D*—H···*A*	*D*—H	H···*A*	*D*···*A*	*D*—H···*A*
N3—H3*n*···O2^i^	0.86	2.03	2.883 (3)	170
C31—H31···O1^ii^	0.93	2.47	3.160 (4)	131

Symmetry codes: (i) −*x*+1, −*y*, −*z*+2; (ii) −*x*, −*y*, −*z*+1.
